# Comparison of Triglyceride-Glucose Index Indices and Fatty Liver Index in Predicting Metabolic Dysfunction-Associated Fatty Liver Disease: A Cross-Sectional Study Conducted in Vietnam

**DOI:** 10.3390/life15111702

**Published:** 2025-11-03

**Authors:** Linh Nhat Boi Nguyen, Thong Duy Vo

**Affiliations:** 1 Department of Internal Medicine, School of Medicine, University of Medicine and Pharmacy at Ho Chi Minh City, Ho Chi Minh City 71724, Vietnam; 2 Department of Gastroenterology, University Medical Center Ho Chi Minh City, Ho Chi Minh City 71724, Vietnam

**Keywords:** fatty liver, fatty liver index, triglyceride-glucose index, triglyceride glucose-body mass index, triglyceride glucose-waist circumference

## Abstract

**Background and Aims:** Metabolic dysfunction-associated fatty liver disease (MAFLD) is increasingly prevalent and linked to liver and cardiometabolic complications. Although liver biopsy remains the diagnostic gold standard, its invasiveness limits routine use, and imaging modalities show variable accuracy. Non-invasive indices such as triglyceride-glucose (TyG), triglyceride glucose-body mass index (TyG-BMI), triglyceride glucose-waist circumference (TyG-WC), and the fatty liver index (FLI) are recommended for screening, yet their performance in Vietnam remains unclear. This study evaluated and compared these indices in Vietnamese adults. **Methods:** A cross-sectional study was conducted at the Health Screening Department, University Medical Center Ho Chi Minh City (September 2024–January 2025). After exclusions, 290 adults undergoing routine check-ups with abdominal ultrasound were included. Clinical and laboratory data were collected to calculate TyG, TyG-BMI, TyG-WC, and FLI, and their diagnostic performance for MAFLD was compared using logistic regression and receiver operating characteristic (ROC) analysis, with area under the ROC curve (AUROC) and 95% confidence intervals (CIs). **Results:** Of 290 participants, 32.76% were diagnosed with MAFLD. Patients with MAFLD were older, more frequently male, and had higher body mass index (BMI), waist circumference (WC), blood pressure (BP), metabolic comorbidities, and abnormal biochemical parameters compared with non-MAFLD. The highest diagnostic performance was observed with TyG-BMI and FLI, both showing area under the receiver operating characteristic curve (AUROC) = 0.89, followed by TyG-WC (0.88) and TyG (0.82). In gender-stratified analysis, indices performed better in females; TyG-BMI achieved the highest AUROC of 0.91, comparable to FLI (0.90). **Conclusions:** TyG, TyG-BMI, TyG-WC, and FLI demonstrated excellent and comparable diagnostic accuracy for MAFLD, with superior performance in women. These indices represent practical, non-invasive tools for MAFLD screening in both clinical and community settings.

## 1. Introduction

Nonalcoholic fatty liver disease (NAFLD) was first described about four decades ago as hepatic steatosis (HS) in the absence of significant alcohol intake and is now the leading cause of chronic liver disease worldwide [1]. However, its definition as a diagnosis of exclusion, together with potential stigmatization and the lack of emphasis on metabolic dysfunction (MD), prompted the proposal of the MAFLD definition in 2020 [2,3]. The term MAFLD, recently redefined as metabolic dysfunction-associated steatotic liver disease (MASLD), is used herein to reflect the diagnostic criteria applied during the study period [3]. The concept of MAFLD has been endorsed by international organizations, including the Asian Pacific Association for the Study of the Liver (APASL), which has developed specific clinical practice guidelines [4]. Its prevalence is projected to rise alongside obesity, type 2 diabetes mellitus (T2DM), and MD, increasing the burden of liver injury and related conditions such as cardiovascular disease (CVD), chronic kidney disease (CKD), and hepatocellular carcinoma [5]. Early identification and diagnosis are crucial to prevent progression to severe complications.

Liver biopsy remains the gold standard for diagnosing MAFLD, but it is invasive and unsuitable for large-scale screening [6]. Abdominal ultrasound, although widely used as the initial imaging tool for HS, is limited by operator dependence and reduced accuracy in obese patients or those with bowel gas or ascites [7]. Computed tomography (CT) and magnetic resonance imaging (MRI) offer superior accuracy but remain restricted by high cost and limited accessibility [4,8]. The APASL recommends serum biomarkers and scoring systems as alternatives when imaging is unavailable, especially in community settings [4]. Several serological indices have been proposed for assessing hepatic steatosis (HS). Among them, the TyG index and its anthropometric derivatives (TyG-BMI, TyG-WC) are closely associated with insulin resistance (IR) [9]—a key pathogenic mechanism of fatty liver disease [10]—and have also been linked to CVD and CKD [11]. In recent years, TyG and its derivatives have emerged as promising markers for predicting HS, with numerous international studies in both NAFLD and MAFLD populations reporting favorable results [12,13,14,15]. The FLI, introduced by Bedogni et al. in 2006, has also been recommended by several hepatology societies, including the APASL, as an alternative to imaging modalities in resource-limited settings [4,16].

To our knowledge, in Vietnam, research has largely focused on estimating the prevalence of MAFLD and its associated factors. For example, a study by Thong and Quynh in patients with NAFLD demonstrated that elevated ALT was significantly associated with a higher prevalence of advanced fibrosis (F3–F4) compared with normal ALT levels [17]. In addition, Thong and colleagues have conducted several in-depth studies on NAFLD, including assessments of factors associated with disease severity and the role of the rs266729 polymorphism in the ADIPOQ gene in its pathogenesis [18,19]. However, the application of serum biomarkers such as TyG, TyG-WC, TyG-BMI, and FLI for predicting fatty liver remains limited in Vietnam. Therefore, this study aims to evaluate and compare the predictive value of TyG and its anthropometrically enhanced versions with that of FLI—an internationally recognized tool—for the prediction of MAFLD in adults. We hypothesized that the triglyceride-glucose-related indices (TyG, TyG-BMI, and TyG-WC) would demonstrate comparable or superior diagnostic accuracy to the fatty liver index (FLI) in predicting MAFLD among Vietnamese adults.

## 2. Methods

### 2.1. Study Design and Setting

This cross-sectional analytical study was conducted at the Health Check-up Center, University Medical Center Ho Chi Minh City, between September 2024 and January 2025. Participants were consecutively recruited from adults attending routine health screening.

Inclusion criteria were age ≥ 18 years and availability of complete anthropometric, biochemical, and ultrasound data.

Exclusion criteria included positive HBsAg or anti-HCV, known liver tumor, significant alcohol consumption, incomplete data, or ongoing treatment for type 2 diabetes mellitus or dyslipidemia.

These criteria ensured a hospital-based but metabolically diverse cohort representing adults attending preventive health examinations. Of the 328 participants initially enrolled, we excluded hepatitis B virus infection (n = 17), hepatitis C virus infection (n = 2), receiving treatment for type 2 diabetes or dyslipidemia (n = 13), incomplete study data (n = 5), and hepatic tumors (n = 1). After these exclusions, a total of 290 participants were included in the final analysis. The study population was categorized into two groups: diagnosed with MAFLD and without MAFLD. A minimum sample size of 270 participants was estimated to achieve 80% power to detect an AUROC ≥ 0.80 with α = 0.05, based on previous studies assessing non-invasive indices for MAFLD prediction. The final sample (n = 290) met this requirement.

### 2.2. Data Collection

A structured questionnaire was used to collect data on age, sex, medical history, and lifestyle factors. Physical activity was defined as ≥150 min/week of moderate intensity, ≥75 min/week of vigorous intensity, or an equivalent combination [20]. Smoking was defined as having smoked ≥100 cigarettes in a lifetime and currently smoking (daily or occasionally) [21]. Alcohol consumption was assessed according to national guidelines [22]. Participants consuming >2 units/day for men, >1 unit/day for women, or drinking on >5 days/week were categorized as having harmful alcohol use and were excluded from MAFLD diagnosis. Participants with occasional or social drinking below these cut-offs were included, consistent with the 2025 APASL guidance that MAFLD and modest alcohol intake may coexist when metabolic dysfunction predominates [22].

Anthropometric measurements included BMI calculated by dividing weight by the square of height in meters (kg/m^2^) [23], WC measured at the midpoint between the lower rib margin and iliac crest or at the umbilical level [24], and BP measured after ≥5 min rest. Hypertension was defined as systolic blood pressure (SBP) ≥ 140 mmHg or diastolic blood pressure (DBP) ≥ 90 mmHg [25].

Fasting (≥8 h) blood samples were analyzed for total cholesterol (TC), low-density lipoprotein cholesterol (LDL-C), high-density lipoprotein cholesterol (HDL-c), triglycerides (TG), aspartate aminotransferase (AST), alanine aminotransferase (ALT), gamma-glutamyl transferase (GGT), fasting plasma glucose (FPG), and glycosylated hemoglobin (HbA1c). Dyslipidemia was defined as having at least one abnormal lipid parameter: TC > 200 mg/dL (5.2 mmol/L), TG > 150 mg/dL (1.7 mmol/L), LDL-C > 100 mg/dL (2.58 mmol/L), or HDL-c < 40 mg/dL (1.03 mmol/L) [26]. T2DM was diagnosed when FPG was ≥7.0 mmol/L (126 mg/dL) or HbA1c was ≥6.5% [27].

### 2.3. Indices and Calculations

The TyG index [28] and its related parameters (TyG-BMI, TyG-WC) [29] were calculated using the following formulas:

TyG = ln [TG (mg/dL) × FPG (mg/dL)/2] TyG-BMI = TyG × BMI; TyG-WC = TyG × WC (cm)

The FLI is derived from four parameters: BMI (kg/m^2^), WC (cm), TG (mg/dL), and GGT (U/L). The calculation formula is as follows [16]:

$$FLI=\frac{e^{0.953}\times \log\left(TG\right)+0.139\times BMI+0.718\times \log\left(GGT\right)+0.053\times VB-15.745}{1+e^{0.953}\times \log\left(TG\right)+0.139\times BMI+0.718\times \log\left(GGT\right)+0.053\times VB-15.745}\times 100$$

### 2.4. Diagnostic Criteria for MAFLD

MAFLD was diagnosed when there was evidence of hepatic steatosis (HS) on abdominal ultrasonography performed using the ultrasound system of the University Medical Center Ho Chi Minh City. All examinations were conducted by a single experienced radiologist with over five years of clinical practice and more than 120,000 ultrasound cases. This approach minimized inter-observer variability. However, formal inter-observer reliability was not assessed and is acknowledged as a study limitation. The diagnosis also required at least one of the following three criteria: (1) overweight/ obesity (BMI ≥ 23 kg/m^2^ for Asians); (2) T2DM diagnosed according to international criteria; or (3) evidence of MD in lean subjects defined as the presence of at least two of metabolic risk abnormalities: WC ≥ 90/80 cm in Asian men and women; BP ≥ 130/85 mmHg or specific drug treatment; plasma TG ≥ 150 mg/dL (≥1.70 mmol/L) or specific drug treatment; plasma HDL-c < 40 mg/dL (<1.0 mmol/L) for men and <50 mg/dL (<1.3 mmol/L) for women or specific drug treatment; prediabetes (i.e., FPG 100–125 mg/dL [5.6–6.9 mmol/L], or HbA1c 5.7–6.4%; homeostasis model assessment (HOMA)—IR score ≥ 2.5; plasma high-sensitivity C-reactive protein (hs-CRP) level > 2 mg/L. HOMA-IR, hs-CRP, and 2 h post-load glucose were excluded from the diagnostic criteria because these tests were not included in the standard health check-up package at the University Medical Center Ho Chi Minh City, and hs-CRP was only available in a costly premium package with limited uptake. Although ultrasound reports recorded hepatic steatosis, detailed grading (mild, moderate, severe) was not consistently documented across all participants; hence, MAFLD was analyzed as a binary variable (presence vs. absence). Future studies with standardized ultrasound grading or CAP measurement are needed to validate these findings.

### 2.5. Statistical Analysis

Data were analyzed using Stata, version 17 (StataCorp LLC, College Station, TX, USA). For descriptive statistics, quantitative variables were assessed for normality using the Shapiro–Wilk test. Variables with a normal distribution were presented as mean ± standard deviation (SD), whereas non-normally distributed variables were expressed as median and interquartile range (IQR). Categorical or binary variables were summarized as frequencies (n) and percentages (%).

For analytical statistics, anthropometric, clinical, and laboratory characteristics between participants with and without MAFLD were compared using the Student’s t-test for normally distributed quantitative variables, the Mann–Whitney U test for non-normally distributed variables, and the chi-square test for categorical variables. Logistic regression estimated odds ratios (OR) and 95% confidence intervals (CI) for MAFLD by quartiles of each parameter, using the lowest quartile as reference. Models included: unadjusted; adjusted for age and sex; and fully adjusted for biochemical, clinical, and lifestyle covariates. Multivariate logistic regression analyses were conducted to adjust for potential confounding factors, including age, sex, body mass index (BMI), blood pressure, fasting glucose, triglycerides, and HDL-cholesterol. Three analytical models were constructed: Model 1 (unadjusted), Model 2 (adjusted for age and sex), and Model 3 (fully adjusted for all covariates). The predictive performance of each index for MAFLD was evaluated by calculating the AUROC, along with corresponding sensitivity (SEN) and specificity (SPE) values, positive predictive value (PPV), and negative predictive value (NPV). The optimal cutoff point for each index was determined using the Youden index. AUROC differences were tested using the DeLong method.

## 3. Results

A total of 290 participants were included in the final analysis, comprising 95 individuals with MAFLD (32.76%) and 195 without MAFLD (67.24%). Table 1 summarizes the demographic, anthropometric, lifestyle, clinical, and biochemical characteristics of the study population stratified by MAFLD status. Participants with MAFLD were older (median 40 vs. 37 years, *p* = 0.034) and more frequently male (68.42% vs. 33.85%, *p* < 0.001). They also had higher BMI, WC, and both SBP and DBP. Regarding lifestyle and comorbidities, the MAFLD group showed higher proportions of current smoking, alcohol consumption, hypertension, T2DM, and dyslipidemia. Biochemical parameters demonstrated significantly higher FPG, HbA1c, TC, TG, LDL-C, AST, ALT, and GGT levels in the MAFLD group, whereas HDL-C levels were lower (*p* < 0.001). Consistently, non-invasive indices, including TyG, TyG-BMI, TyG-WC, and FLI, were all higher in participants with MAFLD compared to those without.

Multivariate logistic regression confirmed that the associations remained significant after adjusting for age, sex, BMI, blood pressure, fasting glucose, triglycerides, and HDL-cholesterol. Table 2 presents the odds ratios for MAFLD according to quartiles of TyG, TyG-BMI, TyG-WC, and FLI, with Q1 serving as the reference group (OR = 1). Three analytical models were applied: Model 1 unadjusted, Model 2 adjusted for age and sex, and Model 3 fully adjusted for clinical, biochemical, and lifestyle factors. In Model 1, the risk of MAFLD increased progressively from Q1 to Q4 across all indices. After adjustment for age and sex (Model 2), the associations remained statistically significant. In Model 3, after additional adjustment for potential confounders, the associations persisted: TyG increased from an OR of 7.13 in Q3 to 10.40 in Q4 (*p* < 0.001); TyG-BMI showed a pronounced rise, reaching an OR of 200.80 in Q4; TyG-WC rose to an OR of 98.89 in Q4; and FLI reached an OR of 193.16 in Q4 (*p* < 0.001). Differences in AUROC were tested using the DeLong method. The large odds ratios reflect strong gradient effects between quartiles; multicollinearity was excluded (VIF < 2.0).

**Figure 1:**
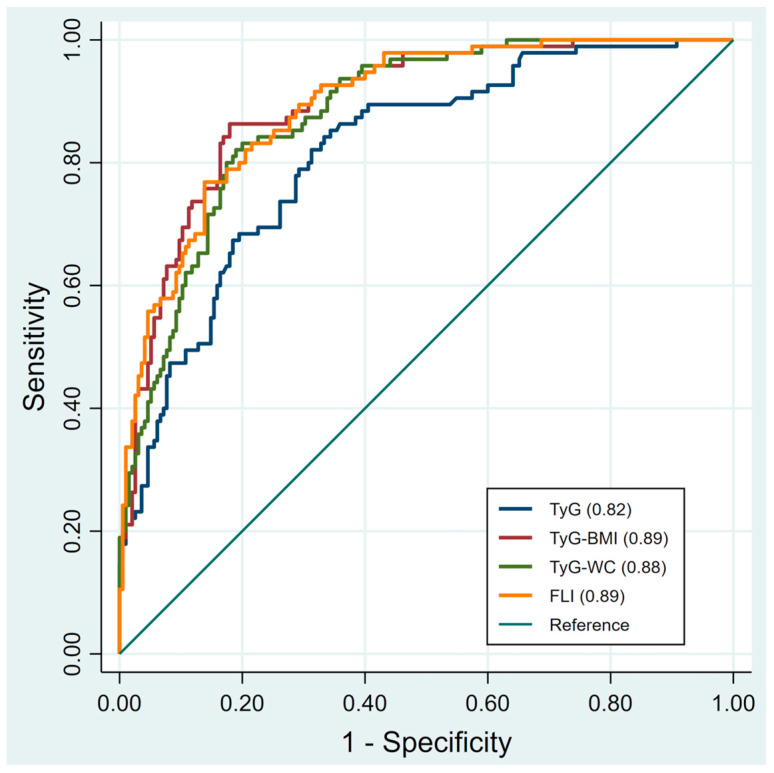
Illustrates the receiver operating characteristics (ROC) curves of each index for predicting MAFLD in all participants. TyG, triglyceride-glucose; TyG-BMI, triglyceride glucose-body mass; TyG-WC, triglyceride glucose-waist circumference; FLI, fatty liver index. Receiver operating characteristic (ROC) curves with 95% confidence intervals (shaded areas) for TyG, TyG-BMI, TyG-WC, and FLI in predicting MAFLD.

Table 3 presents the diagnostic performance of TyG, TyG-BMI, TyG-WC, and FLI in detecting MAFLD. In the overall population, FLI and TyG-BMI demonstrated the highest performance with an AUROC of 0.89 (95% CI: 0.86–0.93). At the cutoff of 37.57, FLI achieved SEN 77% and SPE 86%. TyG-BMI, at the cutoff of 202.85, yielded SEN 86% and SPE 82%. TyG-WC, with a cutoff of 725.13, reached SEN 83% and SPE 80%. Finally, TyG, at the cutoff of 8.48, provided SEN 85% and SPE 66%. In males, TyG-BMI (cutoff 202.85) and FLI (cutoff 37.62) both achieved the highest diagnostic performance with an AUROC of 0.85. In females, TyG-BMI had the highest diagnostic performance with an AUROC of 0.91 at the cutoff of 205.71 (SEN 80%, SPE 90%), followed by FLI with an AUROC of 0.90 at the cutoff of 12.89 (SEN 93%, SPE 75%). The receiver operating characteristics (ROC) curves of TyG, TyG-BMI, TyG-WC, and FLI for predicting MAFLD are presented in Figure 1 for the overall population, male subgroup, and female subgroup.

## 4. Discussion

MAFLD has emerged as a major global health burden, strongly associated with adverse hepatic and cardiometabolic outcomes [1,5], underscoring the need for early detection and intervention. Although liver biopsy remains the diagnostic gold standard, its invasive nature limits large-scale application [6,7], while imaging modalities such as ultrasound, CT, and MRI are constrained by cost, operator dependence, and limited availability in many healthcare settings [4,8]. Consequently, non-invasive serum-based indices are increasingly recommended for screening, with FLI endorsed by major hepatology societies as a practical alternative when imaging is not feasible [4]. In parallel, the TyG index and its derivatives have gained increasing recognition as reliable predictors of fatty liver [12,13,14,15]. In this context, our study evaluated and compared the diagnostic performance of TyG-related indices with that of FLI for predicting MAFLD. We found that all indices demonstrated good diagnostic accuracy, with AUROC values consistently above 0.80. Notably, TyG-BMI, TyG-WC, and FLI were the top-performing indices, and both TyG-BMI and TyG-WC exhibited comparable accuracy to FLI, with no statistically significant differences.

FLI is one of the most extensively studied non-invasive markers and is recommended for clinical use when imaging modalities are not feasible, owing to its simplicity and reliance on routine parameters such as BMI, WC, TG, and GGT [4,16]. In our study, FLI demonstrated very good diagnostic performance for MAFLD with an AUROC of 0.89 in the overall population; notably, its performance was higher in women (0.90) compared with men (0.85). This finding is consistent with the study by Kim et al., which reported AUROCs of 0.864 in the overall population, 0.895 in women, and 0.807 in men [12]. Similarly, Zou et al., in a Western China cohort, observed an AUROC of 0.879 in the general population and also highlighted higher accuracy in women [13]. These results collectively reinforce that FLI provides stable diagnostic accuracy across different populations, with particularly strong performance in women.

TyG index is a simple surrogate marker of IR, which represents the central pathophysiological mechanism of MAFLD [9,10]. TyG comprises TG and FPG, both of which play critical roles in the development and progression of fatty liver [10,28]. Elevated TG reflects lipid metabolism disorders, whereas increased FPG indicates IR and impaired glucose tolerance [10]. This combination makes TyG a reliable indirect marker for detecting MAFLD. In our study, TyG demonstrated moderate diagnostic performance with an AUROC of 0.82, similar to the findings of Kim et al. [12]. (0.79). However, its specificity was relatively limited (66%), suggesting that TyG alone may not fully capture the complexity of the underlying metabolic dysfunction in fatty liver. When anthropometric factors were added, TyG-BMI and TyG-WC showed markedly superior diagnostic performance compared with TyG alone [12,13,14,15]. The addition of BMI reflects overall obesity, while WC represents central obesity; both are strongly associated with IR and hepatic fat accumulation [30].

In our study, TyG-BMI achieved an AUROC of 0.89 in the overall population, 0.85 in men (equivalent to FLI), and 0.91 in women (the highest among all indices). TyG-WC also performed well, with AUROCs of 0.88 in the overall population, 0.83 in males, and 0.88 in females. Thus, both TyG-BMI and TyG-WC demonstrated diagnostic performance comparable to FLI, with even higher AUROCs in women. These results are consistent with Kim’s study, which reported AUROCs of 0.867 for TyG-BMI (0.812 in males, 0.895 in females) and 0.862 for TyG-WC (0.810 in men, 0.894 in women), comparable to those of FLI (0.864 overall, 0.807 men, 0.895 women) [12]. Zou et al., analyzing NHANES data, found AUROCs of 0.85 for TyG-BMI and 0.863 for TyG-WC, while in the Western China cohort, TyG-BMI achieved 0.903 and TyG-WC 0.873, values that were comparable to or even higher than FLI [13]. In addition, Khamseh et al. demonstrated that both indices were strongly associated with NAFLD in overweight and obese individuals and could reliably predict disease risk [29]. Xue reported that TyG-WC (AUROC 0.832) and TyG-BMI (0.822) were the strongest predictors of NAFLD, MAFLD, and moderate-to-severe fibrosis in the U.S. adult population [14]. Similarly, Song showed that TyG-BMI (AUROC 0.827; 0.774 in men; 0.832 in women) and TyG-WC (0.832; 0.783 in men; 0.840 in women) both had good diagnostic value in the Korean population [15]. These findings suggest that TyG-BMI and TyG-WC may serve as reliable alternatives to FLI for predicting MAFLD across diverse populations.

Our study has several notable strengths. First, it is one of the earliest studies in Vietnam to comprehensively compare the diagnostic value of the TyG index and its derivatives (TyG-BMI, TyG-WC) with FLI in predicting MAFLD. Second, the study was conducted on a sufficiently large sample size with complete clinical, anthropometric, and biochemical data, ensuring the reliability of the analyses. Third, we assessed diagnostic performance stratified by sex, thereby highlighting gender-related differences in the utility of these indices. Finally, we applied robust statistical methods, including ROC analysis, quartile-based risk assessment, and AUROC comparisons, which enhance the objectivity of our findings and allow meaningful comparisons with international studies.

This study has several limitations. First, the diagnosis of MAFLD was based on abdominal ultrasonography, which is less sensitive for detecting mild steatosis than FibroScan, CT, or MRI, potentially leading to underestimation of disease prevalence. Second, the cross-sectional design precludes causal inference and long-term prognostic evaluation. Third, because participants were predominantly healthy, working-age adults undergoing routine health check-ups, the findings may not be generalizable to other demographic or high-risk populations. Fourth, noninvasive fibrosis markers such as APRI, FIB-4, or ultrasound elastography were not routinely available in the dataset and therefore were not analyzed. Future prospective studies should integrate these parameters to evaluate the combined diagnostic value of metabolic and fibrosis indices. Fifth, the exclusion of participants receiving treatment for type 2 diabetes mellitus or dyslipidemia may have introduced selection bias toward metabolically healthier individuals, potentially underestimating the true prevalence of MAFLD. Sixth, detailed ultrasound grading of steatosis and inter-observer reliability assessment were not performed. Finally, the reliance on AUROC comparisons without multivariable predictive modeling may limit clinical interpretability. Future studies should validate these indices using integrated predictive models incorporating metabolic and fibrosis-related parameters.

## 5. Conclusions

In summary, the TyG, TyG-BMI, TyG-WC, and FLI indices all demonstrated good diagnostic performance for MAFLD, with AUROC values above 0.80. Among them, TyG-BMI, TyG-WC, and FLI were the best-performing indices, showing comparable accuracy. Importantly, these indices achieved higher diagnostic performance in women. Taken together, the findings support the use of TyG-BMI and TyG-WC as reliable alternatives to FLI for screening and predicting MAFLD, particularly in primary care and community-based settings.

## Figures and Tables

**Table 1:** Baseline characteristics of research participants.

Variables	Total (n = 290)	Non-MAFLD (n = 195)	MAFLD (n = 95)	*p* Value
**Demographic parameters**				
Age (years)	39 (33–47)	37 (32–45)	40 (34–48)	0.034
Gender, n (%)				
Female	159 (54.83)	129 (66.15)	30 (31.58)	<0.001
Male	131 (45.17)	66 (33.85)	65 (68.42)	
**Anthropometric parameters**				
BMI (kg/m^2^)	22.86 (21.08–25.40)	22.10 (20.03–23.44)	25.76 (24–28.57)	<0.001
Waist circumference (cm)	82 (76–87)	79 (74–84)	87 (83–95)	<0.001
SBP (mmHg)	124 (115–133)	120 (111–130)	132 (123–138)	<0.001
DBP (mmHg)	80 (74–88)	79 (70–85)	85 (80–89)	<0.001
**Lifestyle factors**				
Current smoker, n (%)	34 (11.72)	17 (8.72)	17 (17.89)	0.023
Alcohol consumption, n (%)	85 (29.31)	48 (24.62)	37 (38.95)	0.012
Regular exercise, n (%)	203 (70)	131 (67.18)	72 (75.79)	0.133
**Medical history**				
Hypertension, n (%)	46 (15.86)	21 (10.77)	25 (26.32)	0.001
T2DM, n (%)	4 (1.38)	0 (0)	4 (4.21)	0.004
Dyslipidemia, n (%)	270 (93.10)	176 (90.26)	94 (98.95)	0.006
**Serum test**				
FPG (mg/dL)	92 (88–97)	90 (86–96)	96 (90–103)	<0.001
HbA1c (%)	5.49 (5.2–5.75)	5.4 (5.13–5.62)	5.67 (5.4–5.93)	<0.001
Total cholesterol (mg/dL)	197 (178–228)	193 (174–216)	208 (185–239)	0.001
Triglyceride (mg/dL)	108.5 (72–168)	90 (63–128)	168 (114–253)	<0.001
HDL-C (mg/dL)	50 (43–59)	53 (45–61)	44 (40–52)	<0.001
LDL-C (mg/dL)	134.84 ± 30.46	130.63 ± 30.34	143.49 ± 28.99	<0.001
AST (U/L)	22 (18–26)	21 (17–24)	25 (20–31)	<0.001
ALT (U/L)	17 (12–28)	14 (10–23)	27 (18–40)	<0.001
GGT (U/L)	27 (16–52)	21 (13–34)	51 (35–82)	<0.001
**Indices**				
TyG index	8.49 (8.08–8.99)	8.33 (7.94–8.71)	8.99 (8.62–9.45)	<0.001
TyG-BMI	197.08 (173.13–222.61)	182.64 (163.63–199.64)	231.10 (211.58–258.80)	<0.001
TyG-WC	698.61 (626.18–776.16)	647.43 (600.28–711.49)	783.70 (741.51–852.91)	<0.001
FLI	21.05 (8.10–49.10)	10.98 (5.67–25.58)	57.75 (38.17–78.08)	<0.001

MAFLD, metabolic dysfunction-associated fatty liver disease; BMI, body mass index; SBP, systolic blood pressure; DBP, diastolic blood pressure; T2DM, type 2 diabetes mellitus; FPG, fasting plasma glucose; HbA1c, glycosylated hemoglobin; HDL-C, high-density lipoprotein cholesterol; LDL-C, low-density lipoprotein cholesterol; AST, aspartate transferase; ALT, alanine transferase; GGT, gamma-glutamyl transferase; TyG, triglyceride-glucose; TyG-BMI, triglyceride glucose-body mass; TyG-WC, triglyceride glucose-waist circumference; FLI, fatty liver index.

**Table 2:** Odds ratios for MAFLD according to the quartiles of each parameter.

		Model 1			Model 2			Model 3		
		OR	95% CI	*p* Value	OR	95% CI	*p* Value	OR	95% CI	*p* Value
**TyG**	Q1	1	-	-	1	-	0.205	1	-	-
	Q2	3.11	0.94–10.28	0.063	2.23	0.65–7.70	<0.001	1.64	0.44–6.12	0.465
	Q3	14.23	4.70–43.11	<0.001	9.45	2.96–30.15	<0.001	7.13	2.09–24.40	0.002
	Q4	32.43	10.60–99.26	<0.001	19.67	6.01–64.39	<0.001	10.40	2.95–36.73	<0.001
**TyG-BMI**	Q1	1	-	-	1	-	-	1	-	-
	Q2	10.29	1.27–83.45	0.029	7.78	0.94–64.29	0.057	10.11	1.04–98.62	0.047
	Q3	42.26	5.55–321.75	<0.001	27.85	3.56–217.96	0.002	34.46	3.78–314.08	0.002
	Q4	298.29	38.09–2335.71	<0.001	202.02	25.32–1611.87	<0.001	200.80	20.58–1958.99	<0.001
**TyG-WC**	Q1	1	-	-	1	-	-	1	-	-
	Q2	12.98	1.63–103.44	0.015	11.66	1.45–93.98	0.021	11.23	1.35–93.44	0.025
	Q3	50.23	6.61–381.66	<0.001	42.03	5.37–328.77	<0.001	36.32	4.51–292.35	0.001
	Q4	200.84	26.06–1547.58	<0.001	161.58	19.89–1312.67	<0.001	98.89	11.33–862.92	<0.001
**FLI**	Q1	1	-	-	1	-	-	1	-	-
	Q2	10.29	1.27–83.45	0.029	9.36	1.14–77.07	0.038	7.77	0.91–66.05	0.060
	Q3	50.23	6.61–381.66	<0.001	43.00	5.42–341.03	<0.001	39.90	4.83–329.92	0.001
	Q4	232.94	30.07–1804.23	<0.001	204.79	24.95–1681.01	<0.001	193.16	19.86–1878.79	<0.001

Model 1: unadjusted; model 2: adjusted for age and gender; model 3: adjusted for age, gender, AST, ALT, GGT, SBP, DBP, hypertension, prediabetes/T2DM, dyslipidemia, smoking, and exercise. OR, odds ratios; CI, confidence intervals; TyG, triglyceride-glucose; TyG-BMI, triglyceride glucose-body mass; TyG-WC, triglyceride glucose-waist circumference; FLI, fatty liver index.

**Table 3:** Diagnostic performance of selected parameters for predicting MAFLD.

Index	AUROC	95% CI	Cutoff Value	SEN (%)	SPE (%)	PPV (%)	NPV (%)	*p* Value
**All participants**								
TyG index	0.82	0.77–0.87	8.48	85	66	54.7	90.1	<0.001
TyG-BMI	0.89	0.86–0.93	202.85	86	82	70.1	92.5	0.839
TyG-WC	0.88	0.84–0.92	725.13	83	80	66.9	90.7	0.167
FLI	0.89	0.86–0.93	37.57	77	86	73	88.4	-
**Male**								
TyG index	0.73	0.65–0.82	8.80	74	64	66.7	71.2	<0.001
TyG-BMI	0.85	0.79–0.92	202.85	89	68	73.4	86.5	0.923
TyG-WC	0.83	0.77–0.90	728.52	91	64	71.1	87.5	0.393
FLI	0.85	0.79–0.91	37.62	86	71	74.7	83.9	-
**Female**								
TyG index	0.83	0.75–0.91	8.45	83	72	41.0	94.9	0.054
TyG-BMI	0.91	0.86–0.96	205.71	80	90	64.9	95.1	0.431
TyG-WC	0.88	0.83–0.94	685.80	90	79	50.0	97.1%	0.282
FLI	0.90	0.84–0.95	12.89	93	75	46.7	98.0	-

*p* values were calculated using the DeLong test to compare the AUROCs of TyG, TyG-BMI, and TyG-WC with that of FLI. AUROC, area under the receiver operating characteristic curve; CI, confidence intervals; SEN, sensitivity; SPE, specificity; PPV, positive predictive value; NPV, negative predictive value; TyG, triglyceride-glucose; TyG-BMI, triglyceride glucose-body mass; TyG-WC, triglyceride glucose-waist circumference; FLI, fatty liver index.

## Data Availability

The data and code supporting the findings of this study are available from the corresponding author upon reasonable request. We are committed to transparency and scientific integrity, and the data can be shared with qualified researchers for legitimate academic purposes.

## List of Abbreviations

MAFLD, metabolic dysfunction-associated fatty liver disease; TyG, triglyceride-glucose; TyG-BMI, triglyceride glucose-body mass index; TyG-WC, triglyceride glucose-waist circumference; FL, fatty liver index; BMI, body mass index; WC, waist circumference; BP, blood pressure; AUROC, area under the receiver operating characteristic curve; NAFLD, nonalcoholic fatty liver disease; HS, hepatic steatosis; MD, metabolic dysfunction; APASL, Asian Pacific Association for the Study of the Liver; T2DM, type 2 diabetes mellitus; CVD, cardiovascular disease; CKD, chronic kidney disease; CT, computed tomography; MRI, magnetic resonance imaging; IR, insulin resistance; SBP, systolic blood pressure; DBP, diastolic blood pressure; TC, total cholesterol; LDL-C, low-density lipoprotein cholesterol; HDL-c, high-density lipoprotein cholesterol; TG, triglycerides; AST, aspartate aminotransferase; ALT, alanine aminotransferase; GGT, gamma-glutamyl transferase; FPG, fasting plasma glucose; HbA1c, glycosylated hemoglobin; HOMA, homeostasis model assessment; hs-CRP, plasma high-sensitivity C-reactive protein; SD, standard deviation; IQR, interquartile range; OR, odds ratios; CI, confidence intervals; SEN, sensitivity; SPE, specificity; PPV, positive predictive value; NPV, negative predictive value; ROC, receiver operating characteristics.
